# Replenishing NAD^+^ content reduces aspects of striated muscle disease in a dog model of Duchenne muscular dystrophy

**DOI:** 10.1186/s13395-023-00328-w

**Published:** 2023-12-04

**Authors:** Déborah Cardoso, Inès Barthélémy, Stéphane Blot, Antoine Muchir

**Affiliations:** 1 Center of Research in Myology, Institute of Myology, INSERM, Sorbonne University, 75013 Paris, France; 2 “Biology of the Neuromuscular System” Team, U955 IMRB, INSERM, Univ Paris-Est Créteil, 94010 Créteil, France; 3 École Nationale Vétérinaire d’Alfort, IMRB, 94700 Maisons-Alfort, France

**Keywords:** Duchenne muscular dystrophy, GRMD, NAD^+^, Nicotinamide

## Abstract

Duchenne muscular dystrophy (DMD) is an X-linked disease caused by mutations in *DMD* gene and loss of the protein dystrophin, which ultimately leads to myofiber membrane fragility and necrosis, with eventual muscle atrophy and contractures. Affected boys typically die in their second or third decade due to either respiratory failure or cardiomyopathy. Among the developed therapeutic strategies for DMD, gene therapy approaches partially restore micro-dystrophin or quasi-dystrophin expression. However, despite extensive attempts to develop definitive therapies for DMD, the standard of care remains corticosteroid, which has only palliative benefits. Animal models have played a key role in studies of DMD pathogenesis and treatment development. The golden retriever muscular dystrophy (GRMD) dog displays a phenotype aligning with the progressive course of DMD. Therefore, canine studies may translate better to humans. Recent studies suggested that nicotinamide adenine dinucleotide (NAD^+^) cellular content could be a critical determinant for striated muscle function. We showed here that NAD^+^ content was decreased in the striated muscles of GRMD, leading to an alteration of one of NAD^+^ co-substrate enzymes, PARP-1. Moreover, we showed that boosting NAD^+^ content using nicotinamide (NAM), a natural NAD^+^ precursor, modestly reduces aspects of striated muscle disease. Collectively, our results provide mechanistic insights into DMD.

**Supplementary Information:**

The online version contains supplementary material available at 10.1186/s13395-023-00328-w.

## Introduction

Duchenne muscular dystrophy (DMD) is the most common inherited muscular disease affecting one in 3500 live-born males. It is caused by an inherited or spontaneous X-linked mutation in the *DMD* gene [18], leading to a loss of dystrophin expression [12]. As structural and mechanical sarcolemma protein, the absence of dystrophin results in a decrease of muscular fibers stress resistance [25]. During contraction, muscle fibers membrane breaks, and intracellular signaling defects appear [7]. Clinically, first symptoms appear during the first decade of life with delayed acquisition of ambulation, respiratory insufficiency, and cardiomyopathy [22] resulting in an average lifespan of 24 years, which can reach 40 years for patients using ventilatory support [26, 33]. Although progress has been made to identify pathological mechanisms and develop innovative therapeutic strategies, there are currently no optimal therapies. Today, several therapeutic approaches to cure DMD are under investigation, the most promising being gene therapy strategies [24]. However, it is now widely recognized that increased efficacy will likely be obtained with combinatory approaches [8]. Thus, there remains a major unmet medical need for new therapeutic interventions that could be proposed as combo with gene therapy.

Striated muscles convert chemical energy from adenosine triphosphate (ATP) into mechanical activity, which is necessary to generate movement and maintain posture. Therefore, striated muscles are important metabolic tissues able to store, produce, and consume substrates such as amino acids and carbohydrates contributing to provide energy needed during physical activity [9]. Given that striated muscles are metabolically important tissues due to a vital role of nicotinamide adenine dinucleotide (NAD) in energy production, NAD emerges as potential new therapeutic target for muscular dystrophies [10]. NAD is a metabolic cofactor found in cells either in its oxidized (NAD^+^) or reduced (NADH) form [3]. Poly(ADP-ribose) polymerases (PARPs) are major cellular NAD^+^ consumers that have key cellular functions such as response to cellular stress [13]. Considering the key role of NAD in different cellular processes, maintaining the balance between NAD^+^ consumption and biosynthesis is extremely important for cellular homeostasis and tissue function. Three independent intracellular pathways allow NAD^+^ synthesis from various precursors. De novo and Preiss-Handler pathways lead to NAD^+^ synthesis from tryptophan (Trp) and nicotinic acid (NA) in the diet, but the most important pathway for maintaining intracellular NAD^+^ levels is the salvage pathway [28]. It exists another NAD^+^ precursor—nicotinamide riboside (NR)—which enhances NAD^+^ levels through the salvage pathway when catalyzed by nicotinamide riboside kinase (Nmrk) [4, 27]. In the salvage pathway of NAD^+^ biosynthesis, nicotinamide phosphoribosyltransferase (Nampt) was identified as a rate-limiting enzyme that converts NAM to nicotinamide mononucleotide (NMN) [11]. NAD^+^ is then synthesized from NMN-by-NMN adenylyltransferase (Nmnat) [31]. NAD^+^ functions as a cofactor for a multitude of enzymatic reactions within different cellular compartments [14]. NAD^+^ functions have expanded beyond its role as a coenzyme, as NAD^+^ also acts as degradation substrates for a wide range of enzymes, such as sirtuins and poly(ADP-ribose) polymerase (PARPs) [3]. PARPs are nuclear enzymes of eukaryotic cells that participate in DNA repair in response to genotoxic stress, [2, 20]. When activated by DNA breaks, PARPs initiate an energy-consuming cycle by transferring ADP-ribose units from NAD^+^ to nuclear proteins. This process results in rapid depletion of the intracellular NAD^+^ pools, slowing the rate of glycolysis and mitochondrial respiration, eventually leading to cellular dysfunction and death.

Previous studies have shown that muscles NAD^+^ levels are low in aging and many pathological conditions (reviewed in [16]). In muscles of mdx mouse model of DMD, decreased NAD^+^ levels have been observed [29]. Our study reports a decrease of NAD^+^ content in the golden retriever muscular dystrophy (GRMD) dog model of DMD, which is known to more faithfully reproduce the human disease course than other available animal models. Moreover, we showed that short-term NAM supplementation is able to boost NAD ^+^ content in skeletal muscle and to slow the course of the disease.

## Results

### GRMD dogs display an alteration in NAD^+^ levels

The GRMD dog represents a translational bridge between mice and humans, as it mimics more closely the human disease than other existing mammalian models of dystrophin deficiency (Figure S1A) [12]. GRMD dogs share the same pathogenesis as human disease [19] with more severe phenotype than *mdx* mouse considered therefore as more relevant model for identification of therapeutic strategies. GRMD dogs harbor a mutation in the dystrophin gene resulting in a loss of dystrophin protein in striated muscles (Figure S1B) and display dystrophic muscle lesions, progressive fibrosis (Figure S1C–F), early locomotor impairment, and premature death due to respiratory or cardiac failure.

**Fig. 1:**
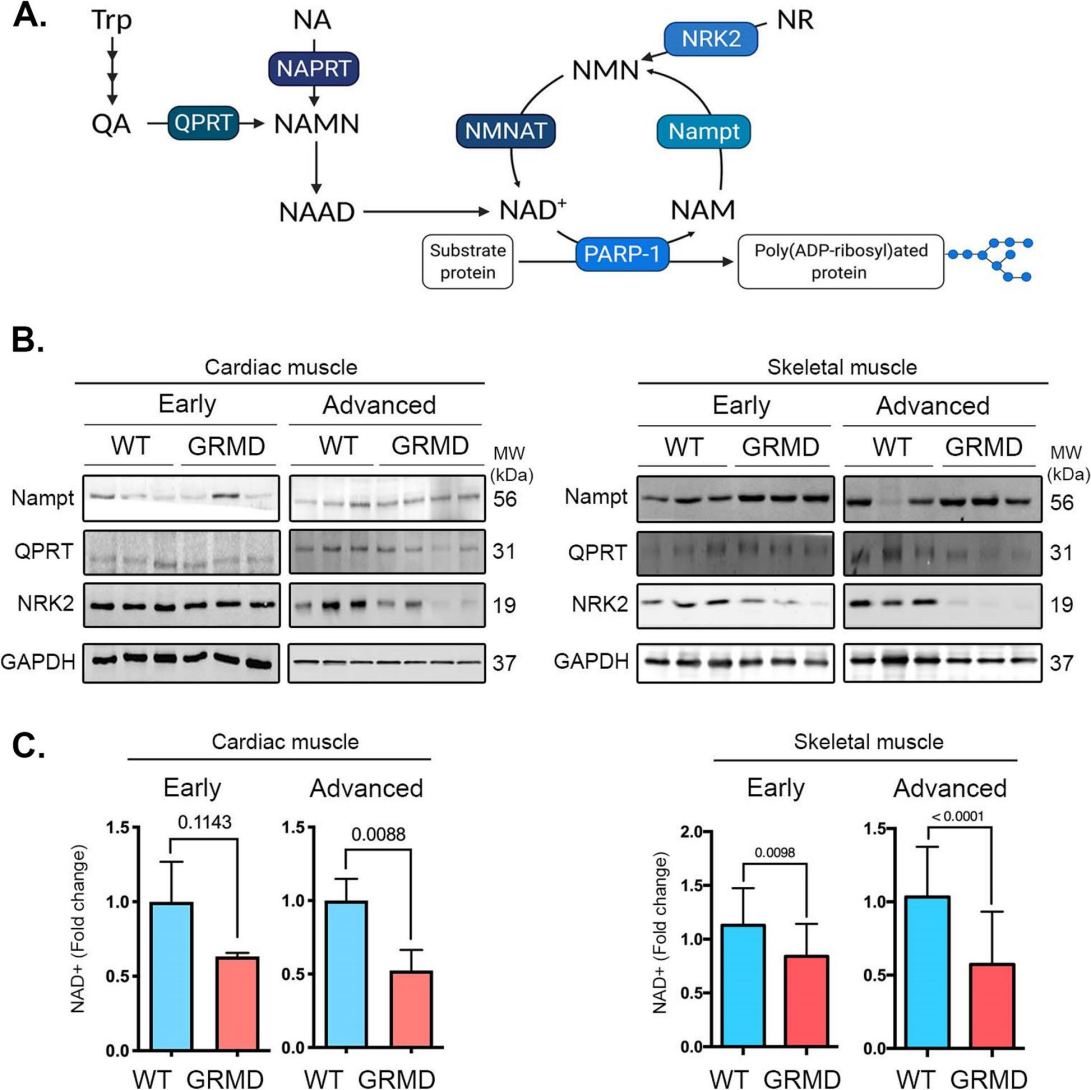
NAD^+^ salvage pathway is altered in striated muscles of golden retriever muscular dystrophy. **A** Schematic representation of the NAD^+^ biosynthesis. Created with BioRender.com. **B** Immunoblots showing Nampt, QPRT, and NRK2 protein levels in cardiac and skeletal from early and advanced age wild-type dogs (*n* = 3) and GRMD (*n* = 3) dogs. GAPDH was used for normalization. **C** Quantification of cardiac and skeletal muscle NAD^+^ content (fold change) in wild-type dogs at early and advanced age (*n* = 4) and GRMD dogs at early and advanced disease (*n* = 4). Bars indicate mean ± standard error of mean, and numbers above columns indicate *P*-values. Differences were analyzed by unpaired *t*-test

To evaluate the relationship between NAD metabolism and DMD, we first assessed the expression of NAD^+^ salvaging and NAD^+^ consuming enzymes (Fig. 1A) in striated muscles from GRMD dogs, along the progression of the disease. There was a decreased expression of QPRT and NRK2 salvaging enzymes in both cardiac and skeletal muscles from GRMD dogs compared with WT dogs (Fig. 1B). Nampt expression was unchanged in both cardiac and skeletal muscles from GRMD dogs compared with WT dogs (Fig. 1B). Given that a consequence of altered NAD^+^ salvage pathways would lead to a drop in cellular NAD^+^ level, we next assessed NAD^+^ level in GRMD striated muscle. We showed that the NAD^+^ content was significantly lowered in heart and skeletal muscles from GRMD dogs with advanced disease compared with age-matched adult WT dogs (Fig. 1C). The decrease of NAD^+^ content was an early finding, being evidenced in tissues from young animals at early disease stages both in cardiac and skeletal muscles. An aggravation of this decrease was found in tissues originating from dogs at advanced disease stages, suggesting a correlation between altered cardiac and skeletal muscle structure and tissular NAD^+^ content (Figure S1).

**Fig. 2:**
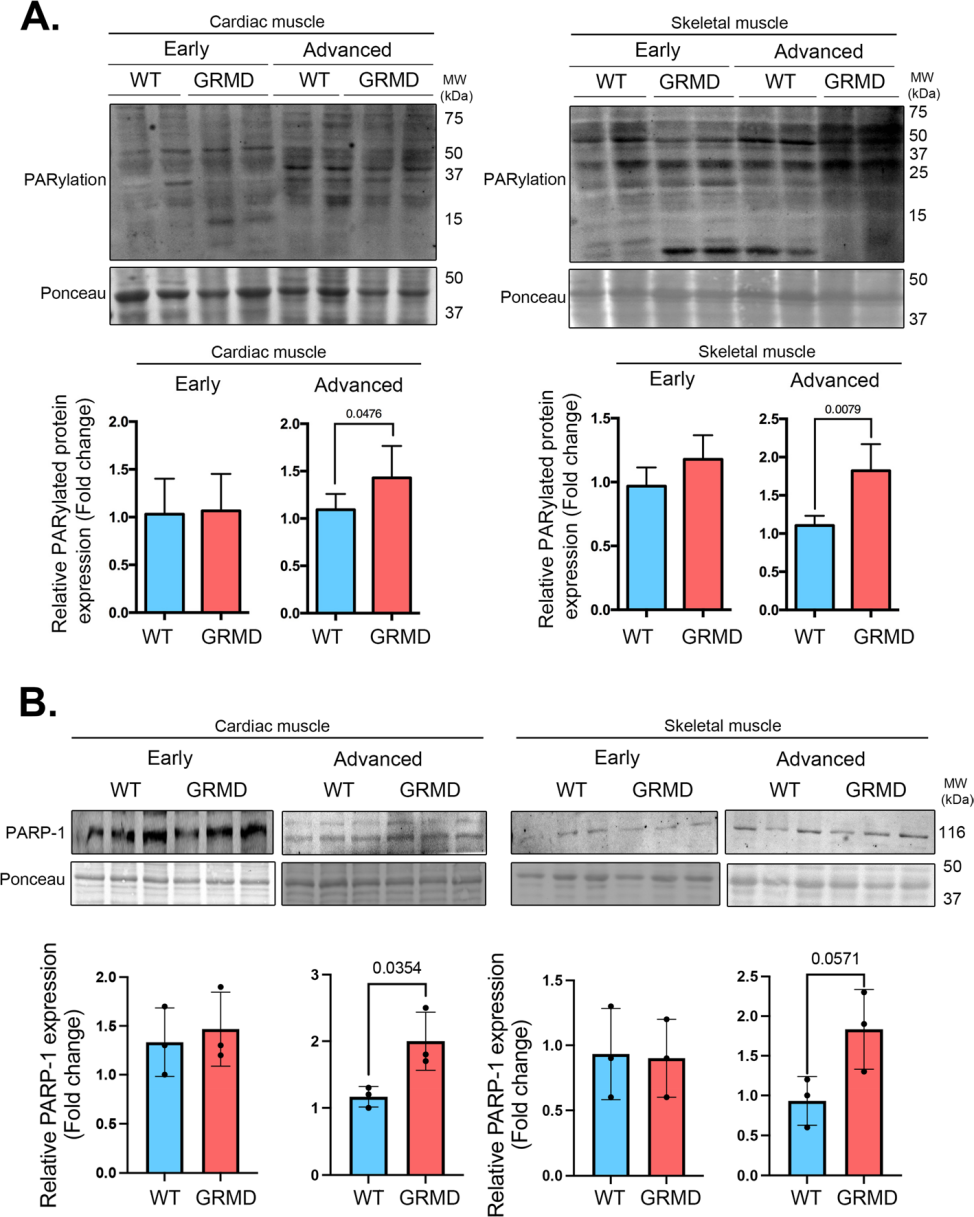
Global poly(ADP-ribosylation) is increased in striated muscles of golden retriever muscular dystrophy. **A** Representative immunoblots showing total PARylated protein level expression with PAR antibody in cardiac and skeletal muscle from early and advanced age WT and GRMD dogs. Ponceau was shown as loading control. Bars indicate mean ± standard error of mean (relative to ponceau), and numbers above columns indicate *P*-values (*n* = 3 dogs for each condition). Differences were analyzed by unpaired *t*-test. **B** Representative immunoblots showing PARP-1 protein level expression in cardiac and skeletal muscle from early and advanced age WT and GRMD dogs. Ponceau was shown as loading control. Bars indicate mean ± standard error of mean (relative to ponceau), and numbers above columns indicate *P*-values (*n* = 3 dogs for each condition). Differences were analyzed by unpaired *t*-test

Given that PARPs are major cellular NAD^+^ consumers, we then asked the consequences of decreased skeletal muscles NAD^+^ on PARP-1 activities. PARPs enzymes bind and cleave NAD^+^ to produce NAM and ADP-ribose and couple one or more ADP-ribose units to promote PARylation of acceptor proteins. The level of PARylation detected by poly(ADP-ribose) (PAR) monoclonal antibody was increased in both cardiac and skeletal muscles from GRMD dogs presenting an advanced disease compared with age-matched WT dogs (Fig. 2A). This is correlated with an increased PARP-1 expression level in both cardiac and skeletal muscles from GRMD dogs presenting an advanced disease (Fig. 2B). Taken together, these findings suggest that increased PARylation and altered NAD^+^ salvage pathways are involved in striated muscle NAD^+^ depletion in GRMD dogs.

### In vivo NAM treatment restores NAD^+^ level

**Fig. 3:**
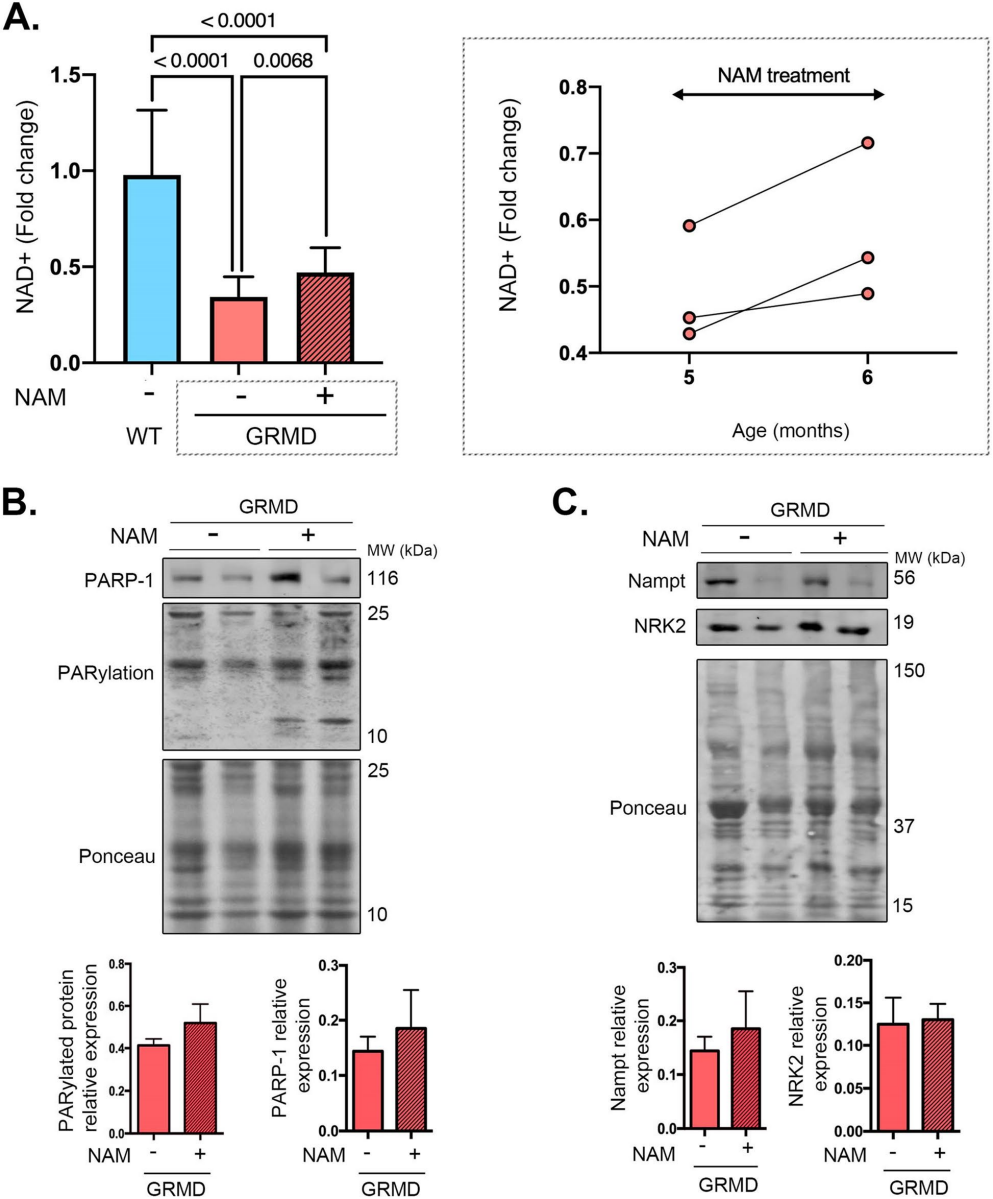
NAM supplementation improves NAD^+^ levels in striated muscles of golden retriever muscular dystrophy. **A** Quantification of skeletal muscles NAD.^+^ content from WT (*n* = 4), GRMD (*n* = 3), and NAM-treated GRMD (*n* = 3) dogs. Bars indicate means ± standard errors of means, and numbers above columns indicate *P*-values. Differences were analyzed by one-way ANOVA. **B** Immunoblots showing total PARylated protein level expression with PAR antibody in skeletal muscle from GRMD (*n* = 2) dogs treated or not with NAM. Ponceau was shown as loading control. The bar graphs represent PARylated protein relative expression (mean ± standard error of means). **C** Representative immunoblots showing Nampt and NRK2 protein level expression in skeletal muscle from GRMD dogs treated or not with NAM. Ponceau was shown as loading control. The bar graphs represent PARP-1 and Nampt protein relative expression (mean ± standard error of means) (*n* = 3 dogs for each condition)

Given that there was a decrease of NAD^+^ level in both cardiac and skeletal muscles from GRMD dogs, and a decreased NRK2 enzyme expression predicting a low NAD^+^ boosting effect of NR supplementation, we next tested whether NAM, the substrate for Nampt (Fig. 1A), could increase NAD^+^ content and therefore improve muscular function. GRMD dogs were treated with NAM (500 mg. 2/day), starting at 5 months of age for 1-month duration. At the end of the treatment, the NAD^+^ level was increased in skeletal muscles from treated GRMD dogs relative to their baseline value (Fig. 3A). NAM-treated dogs exhibited an increased PARylation and PARP-1 expression compared to before treatment (Fig. 3B). Moreover, feeding GRMD dogs with NAM led to increase in Nampt expression, while NRK2 expression was unchanged (Fig. 3C). These results suggest that NAM effectively increased NAD^+^ level in GRMD dogs despite increased PARylation.

### In vivo NAM treatment modestly improves skeletal muscle function

**Fig. 4:**
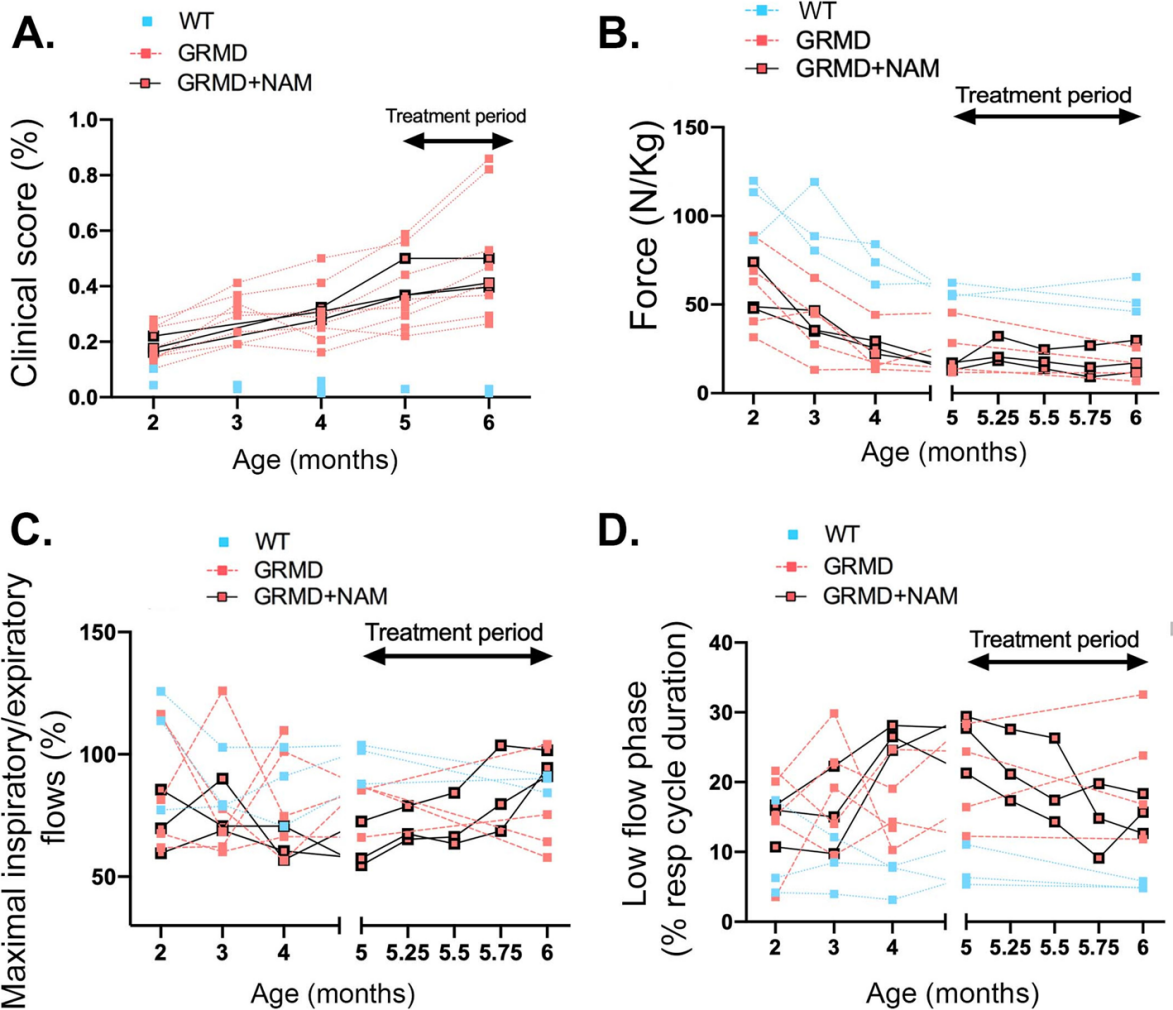
NAM supplementation stabilized muscular function of golden retriever muscular dystrophy. **A** Clinical score assessed monthly in the three treated dogs (*n* = 3), relative to WT (*n* = 3), and untreated GRMD dogs (*n* = 8). **B** Force production during gait measured by 3D accelerometry: longitudinal follow-up in the three treated dogs during a 3-month-period before treatment (*n* = 3) and during treatment, relative to age matches WT (*n* = 3), and untreated GRMD dogs (*n* = 5 at inclusion, *n* = 4 from 5 months of age). **C** Percentage of maximal respiratory and expiratory flows assessed monthly in the three treated dogs (*n* = 3), relative to WT (*n* = 3), and untreated GRMD dogs (*n* = 4). **D** Percentage of low-flow phase assessed monthly in the three treated dogs (*n* = 3), relative to WT (*n* = 3), and untreated GRMD dogs (*n* = 4)

These data led us to test whether boosting NAD^+^ content using NAM delays the progression of striated muscle dysfunction in DMD. We determined whether a short-term treatment could improve function. No treatment effect on skeletal muscle force generation and half-relaxation time from GRMD dogs was detected (Figures S2). From a more global point of view, a trend towards a drift from the natural disease trajectory was observed in the NAM-treated dogs. The three treated dogs tended to stabilize their clinical score relative to untreated GRMD dogs, though the difference was not statistically significant (*P* = 0.127) (Fig. 4A). Gait analysis revealed enhanced force in one animal at the end of the treatment period and a slight decrease in the two others, while untreated GRMD dogs tended to have a more pronounced decrease of the gait force within the same period of time (*P* = 0.078) (Fig. 4B). We showed that the NAM treatment was not able to restore neither the muscle structure nor serum biomarkers levels (creatine kinase and myostatin) in GRMD dogs (Figures S3). This could be due to the short duration of the treatment. Respiratory function analysis showed a significant improvement in the peak inspiratory/expiratory flows ratio during the treatment, relative to untreated GRMD dogs within the same period of time (*P* = 0.035) (Fig. 4C). The low-flow phase also nearly significantly improved in treated GRMD dogs compared with untreated GRMD dogs (*P* = 0.059) (Fig. 4D). Left ventricular shortening fraction remained within normal values in the three dogs during the treatment period, consistently with the well-described delay between skeletal muscle and cardiac function involvement in this model. Thus, short-term NAM treatment leads to a mild beneficial effect in GRMD dogs.

## Discussion

**Fig. 5:**
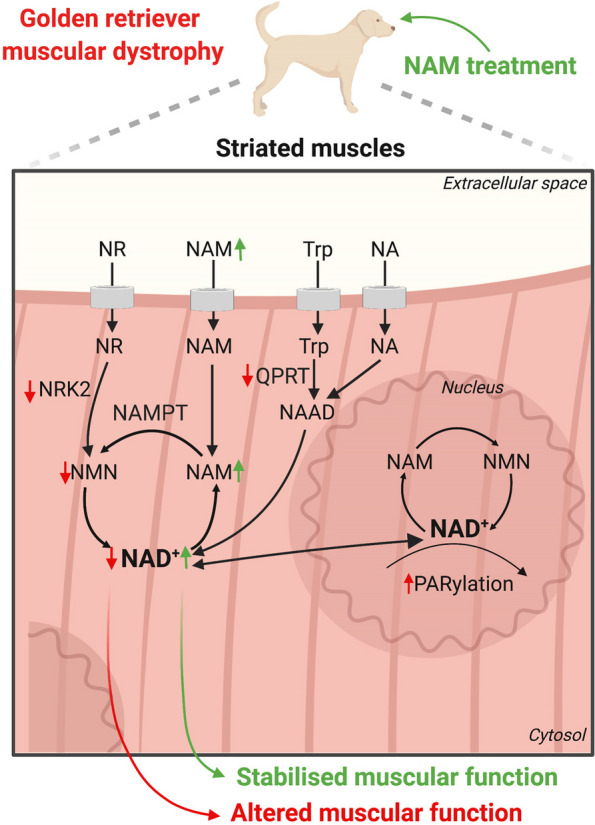
Working model. Schematic representation of the mechanism of NAD^+^-mediated alteration of muscle performance and therapeutic intervention in GRMD dogs. Created with BioRender.com

Given the importance of NAD^+^ in mitochondria dysfunction, energetic defect, calcium dysregulation, and oxidative stress, we sought to identify mechanisms linking NAD^+^ and striated muscles dysfunction in GRMD dogs as a relevant large animal model of DMD. Previous studies in *mdx* mouse model of DMD identified NAD^+^ as a relevant therapeutic target for DMD [29]. We have demonstrated here that muscular dystrophy in GRMD dogs is associated with muscle NAD^+^ depletion, which can potentially be monitored as an index of disease severity. Reduced NAD^+^ levels are likely the result of PARP activation and alteration of both the de novo and the salvage pathway (Fig. 5) as postulated from the robust QPRT and NRK2 protein level that we observed in both cardiac and skeletal muscles from GRMD dogs. Two complementary strategies exist for manipulating tissue NAD^+^ pools. The first is to enhance NAD^+^ production by supplementing biosynthetic precursors. The second strategy is to inhibit the enzymatic consumption of NAD^+^. Our study therefore demonstrates that boosting NAD^+^ production, using NAM, could be effective to impact on the disease progression in GRMD dogs (Fig. 5). While primary NAD^+^ deficiency drives striated muscle weakness, our current data strongly suggest that NAD^+^ boosting in GRMD skeletal muscles has partial beneficial effects on locomotor and respiratory functions. Increasing the number of animals as well as the treatment duration could be the following step to confirm this solid trend on several independent functional indices. We used NAM, as opposed to NR that has been described in two separated studies [29], given that NRK2 (the enzyme that converts NR into NMN) expression was lowered in striated muscle of GRMD dogs. This could partially explain the lack of benefit observed by Frederick and colleagues in mdx mice.

Recent studies indicate that dietary supplementation with NR protects against high-fat-diet-induced obesity [6] while improving hepatic function and protecting against diabetic neuropathy [32]. Similarly, NMN enhances insulin sensitivity in against high-fat-diet-fed mice [35]. Moreover, these NAD precursors are effective at extending lifespan and reducing metabolic disease [15, 17]. NAM is one of three NAD precursor vitamins that is largely made available for NAD salvage via degradation of dietary NAD. NAM has long been used to treat diverse disease conditions, such as inflammatory diseases and dementia, in humans and animal models [34]. In addition, it has been shown that chronic NAM supplementation improves health span measure [23]. Our study therefore demonstrates that the use of NAM, a nutraceutical that could be easily implemented in DMD patients, could be effective for managing the progression of muscular dystrophy together with the available therapeutic arsenal and future innovative strategies. This will ultimately improve our ability to develop targeted disease-modifying therapy and to define specific subgroups of the DMD patients, which are likely to respond to these treatments. Our observations may have broad implications for other neuromuscular diseases that are characterized by PARP activation.

## Methods

### Animal

All procedures were approved by the Ethical Committee of EnvA, ANSES, and UPEC and by the French Ministry of Research under the respective approval numbers 2021–05-04–19 and 31,081–2019112814263892 and were performed in accordance with the relevant guidelines and regulations. Samples obtained from early disease (younger than 6 months old) and advanced disease (6 months old or older) GRMD and age-matched healthy dogs were used for experiments.

### NAM supplementation

A total of 500 mg of nicotinamide (Nicobion ND) were orally administrated to GRMD dogs twice a day in a piece of palatable food. This dose was within the doses usually administered to dogs with inflammatory dermatological diseases [, 18]. The treatment duration was 1 month starting from 5 months of age, the day after the baseline in vivo functional evaluation tests and muscle biopsies.^1^

### Immunoblotting

Total proteins were isolated by resuspending striated muscle tissues in extraction buffer (Cell Signaling) completed with deacetylation inhibitors cocktail (Santa Cruz Biotechnology). The lysates were separated by 10% SDS–polyacrylamide gel electrophoresis and transferred to nitrocellulose membranes (Invitrogen). Blotted membranes being were blocked in 5% bovine serum albumin (BSA) in Tris-buffered saline containing 1% Tween 20 (TBS-T) for 1 h at room temperature followed by incubation overnight at 4 °C with the appropriate primary antibody (Table S1). After washing with TBS-T, membranes were for 1 h at room temperature incubated with secondary anti-rabbit or anti-mouse antibodies conjugated to a fluorescent dye (Table S1). Subsequent to being washed with TBS-T, the signal was revealed using ChemiDoc MP Imaging System (Biorad). Bands were quantified using ImageJ software. Membranes stained with Ponceau are presented in Figure S4.

### NAD^+^ concentration quantification

Fresh striated muscle tissues were homogenized and processed according to previous report [30]. The concentration of NAD^+^ was determined using a spectrophotometric assay as described previously [5]. Absorption measurements were carried out in 96-well plates by spectrophotometry (Tecan).

### Immunostaining and histology

Tissues were snap-frozen in isopentane cooled in liquid nitrogen and stored at − 80 °C. A 10-µm sections were cut and stained with hematoxylin and eosin or sirius red. For immunostaining, sections were rehydrated in PBS and fixed with acetone-methanol (v:v). Endogen peroxidase activity was blocked by 3% H_2_O_2_, and sections were incubated in primary dystrophin antibody (NCL-Dys2, 1/20 In PBS 10% goat serum) 1h30 at RT. After washing with PBS, samples were incubated with secondary antibody (goat anti-mouse IgG (H + L)-dextran polymer-HRP, Dako) for 30 min at RT. Staining was revealed using DAB (Dako), and counterstaining of nuclei was performed using hematein. Slices were mounted in Canada balsam medium. Immunostaining and stained slices were imaged in bright field under a Leica light microscope.

### Muscle biopsies

Muscle biopsies were performed on dogs under general anesthesia, with a morphine analgesia. Biceps femoris and sartorius cranialis biopsies were taken surgically and immediately snap-frozen in isopentane cooled in liquid nitrogen or placed in 75% EtoH/25% HEPES 10-mM pH 7.1 buffer on ice for subsequent NAD extraction. For NAD^+^ concentration quantification, two skeletal muscles were used, the biceps femoris and the sartorius cranialis muscles, and the samples were categorized into 2 subgroups according to the age of the animals: early disease (< 6 months of age) and advanced disease (≥ 6 months of age). For both skeletal muscles, biopsies from 2 WT and 4 GRMD younger than 6 months of age (2 and 4 months of age), and from 2 WT and 5 GRMD at 6 months of age or older, were used. NAD^+^ concentration was also assessed in left ventricular free wall biopsies (postmortem samples) and subdivided into two subgroups: pre-fibrotic phase (≤ 6 months of age) and fibrotic phase (> 6 months of age). Biopsies from 2 WT and 3 GRMD dogs between 2 and 6 months of age and from 2 WT and 4 GRMD older than 6 months of age (11 to 69 months) were used. The selected age threshold differed between skeletal and cardiac muscle studies, because of the well-described delay in myocardial involvement in this model. Indeed, skeletal muscles from 6-month-old GRMD dogs are already fibrotic, relative to myocardium which is still devoid of fibrosis at this stage. For Western-blot analysis, skeletal muscle (biceps femoris) from early-diseased GRMD dogs and age-matched WT dogs (*n* = 3 each) and from advanced-diseased GRMD dogs and age-matched WT dogs (*n* = 3 each) was used. Left ventricular free wall biopsies from GRMD dogs in the pre-fibrotic phase of the disease and age-matched WT dogs (*n* = 3 each) and from GRMD dogs in the fibrotic phase of the disease and age-matched WT dogs (*n* = 2 each) were used as well.

### Histopathological quantification

Quantitative analyses were performed on hematoxylin and eosin staining. Entire muscle sections were analyzed using Visilog 7.0 software (Noesis). A grid was superimposed onto the image, and muscle morphology was assessed at each of the intercepts and manually annotated (1000 annotations per section). The percentage of endomysium was calculated, as well as the percentage of pathological features not corresponding to normal myofibers, called histopathology index.

### Serum biomarkers

Serum CK activity was measured using a biochemical analyzer (Catalyst, IDEXX) at the age of 6 months on the 3 NAM-treated dogs (end of treatment) and compared to 6 age-matched untreated GRMD dogs and 3 WT dogs. At the same timepoint, myostatin serum concentration was assessed by ELISA as previously described [21], using the Quantikine ELISA kit no. DGDF80 (R&D Systems).

### In vivo force measurement

Dogs under general anesthesia (induction with propofol, maintenance with isoflurane in 100% O_2_) were positioned in dorsal recumbency, and their left hind limb was positioned in a dedicated ergometer. Knee and tarsus joints were maintained 90° flexed, the femur being vertically and the tibia horizontally positioned. The metatarsophalangeal segment was placed vertically and attached to a plate linked to two force transducers. Supramaximal stimulation of the peroneal nerve was performed to obtain twitch or tetanic contraction. Force values were normalized by the length of the tibial segment measured in the ergometer, to obtain relative force values.

### Clinical score

Dogs were clinically assessed using a previously described scoring grid, consisting of 17 items focusing on locomotor, postural, deglutition, and respiratory criteria. Each item was graded from zero (normal situation) to two (worse situation), and the sum of values of all the items was used for analysis and expressed as a percentage of the maximal possible score.

### Gait analysis

Gait quality was assessed using 3D accelerometry as previously described. Briefly, dogs wearing a device containing three orthogonally positioned accelerometers (Locometrix®) were encouraged to run or walk at their preferred gait and speed along a dedicated corridor. Accelerations generated near to the center of gravity at rest, in the three gait axes, were recorded at 100 Hz. Traces were analyzed in the software Equimetrix. Total power was calculated as the sum of the integral of the power spectrum obtained by a fast Fourier transform on the trace obtained in each of the three axes. The force index was calculated by normalizing the total power by the gait speed and was expressed in N/kg.

### Respiratory inductance plethysmography

Respiratory function analysis was performed on awake dogs using a device designed to acquire the stretching signal from a thoracic and an abdominal belt at 100 Hz during tidal breathing (EmkaPACK 4G system, Emka Technologies). The signal was calibrated by a concomitant spirometric recording obtained through a pneumotachometer linked to a facemask. Maximal inspiratory and expiratory flows were calculated in the software ecgAUTO (Emka Technologies) and averaged from curves obtained during steady-state breathing without movement artifacts. The percentage of the respiratory cycle duration spent at a flow lower than 10% the maximal expiratory flow (“low-flow phase”) was also calculated.

### Statistics

Values for immunoblots and NAD^+^ content were compared using an unpaired Student’s *t*-test. Comparisons of striated muscle function parameters between groups of dogs (treated and untreated) was performed on the data at baseline and end treatment (or at 5 and 6 months of age for untreated dogs) using a repeated measures ANOVA, with the age as a within factor and the group as a between factor. Comparisons of histopathology analyses and serum biomarkers at 6 months of age were performed using a one-way ANOVA with groups as a between factor. Statistical analyses were performed using Prism 6.0 software (GraphPad).

### Supplementary Information

**Additional file 1:** **Fig. S1.** Golden retriever model of muscular dystrophy: a more translatable model to human DMD. (A) Representative images of WT and GRMD dogs at 9 months of age. (B) Dystrophin immunostaining on striated muscle sections from WT and GRMD dogs at 6 months of age and of cardiac muscle sections (left ventricular free wall) at 8 months of age (x40). (C) Representative heart sections from pre-fibrotic (6 month-old) or fibrotic (12 month-old) WT and GRMD dogs stained with hematoxylin & eosin (H&E) (x40). (D) Representative skeletal muscle (Biceps femoris) sections from juvenile (6 month-old) or adult (12 month-old) WT and GRMD dogs stained with hematoxylin & eosin (H&E) (x40). (E) Representative pre-fibrotic and fibrotic heart sections (left ventricular free wall) from  GRMD and age-matched WT dogs stained with Sirius red (x40). (F) Representative skeletal muscle (Biceps femoris) sections from early- or advanced-diseased GRMD dogs and age-matched WT stained with sirius red (x40). **Fig. S2.** NAM supplementation modestly improves skeletal muscle function. (A) Relative tetanic force at 50 Hz in the three treated GRMD dogs before and after one month of NAM treatment (*n*=3), relative to age-matched WT (*n*=3) and untreated GRMD dogs (*n*=8). (B) Half-relaxation time after a tetanic contraction measured during in vivo force measurement test, in the three treated dogs (*n*=3), before treatment and after one month of NAM treatment, relative to age-matched WT (*n*=3) and untreated GRMD dogs (*n*=8). **Fig. S3.** Histopathology analysis and serum biomarkers. (A) Percentage of endomysial fibrosis quantified on biceps femoris muscle biopsies from 6 month-old Healthy (*n*=3), GRMD (*n*=7) and NAM-treated GRMD (*n*=3) dogs. ****, *P*-value<0.00005 (B) Histopathology index quantified on biceps femoris muscle biopsies from 6 month-old Healthy (*n*=3), GRMD (*n*=7) and NAM-treated GRMD (*n*=3) dogs. **, *P*-value<0.005 (C) Serum creatine kinase (Ck) measured at 6 months of age in Healthy (*n*=3), GRMD (*n*=6) and NAM-treated GRMD (*n*=3) dogs. **, *P*-value<0.005 (D) Serum myostatin concentration measured at 6 months of age in Healthy (*n*=3), GRMD (*n*=5) and NAM-treated GRMD (*n*=3) dogs. ****, *P*-value<0.00005. **Fig. S4.** Uncropped images of membranes stained with Ponceau. **Table S1.** Antibodies used for western blotting.

## Data Availability

All data supporting the findings of this study are available within the paper and its supplementary information.
