# Supplementary material for: Replenishing NAD+ content reduces aspects of striated muscle disease in a dog model of Duchenne muscular dystrophy
Source: Skelet Muscle. 2023 Dec 4;13:20. doi: 10.1186/s13395-023-00328-w (PMC10694913; doi:10.1186/s13395-023-00328-w)
Supplement: Supplementary file 1 [file 13395_2023_328_MOESM1_ESM.docx]

SUPPLEMENTAL INFORMATION

Tables S1: Antibodies used for western blotting

| Antibody | Provider | Reference | Dilution |
| --- | --- | --- | --- |
| GAPDH (Rabbit) | Abcam | #ab8245 | 1/1,000 |
| Nampt (Rabbit) | Abcam | #ab4580 | 1/1,000 |
| Nrk2 (Rabbit) | US Biological | #037146 | 1/1,000 |
| PAR (Mouse) | EM Millipore | AM80 | 1/1,000 |
| PARP-1(Rabbit) | Santa Cruz Biotechnology | #sc-8007 | 1/1,000 |
| QPRT(Rabbit) | Abcam | #ab171944 | 1/1,000 |
| Goat Anti-Mouse IgG StarBright Blue 520 | Bio-Rad | #12005866 | 1/1,000 |
| Goat Anti-Rabbit IgG StarBright Blue 520 | Bio-Rad | #12004161 | 1/1,000 |

Figure S1: Golden retriever model of muscular dystrophy: a more translatable model to human DMD.

(A) Representative images of WT and GRMD dogs at 9 months of age. (B) Dystrophin immunostaining on striated muscle sections from WT and GRMD dogs at 6 months of age and of cardiac muscle sections (left ventricular free wall) at 8 months of age (x40). (C) Representative heart sections from pre-fibrotic (6 month-old) or fibrotic (12 month-old) WT and GRMD dogs stained with hematoxylin & eosin (H&E) (x40). (D) Representative skeletal muscle (Biceps femoris) sections from juvenile (6 month-old) or adult (12 month-old) WT and GRMD dogs stained with hematoxylin & eosin (H&E) (x40). (E) Representative pre-fibrotic and fibrotic heart sections (left ventricular free wall) from  GRMD and age-matched WT dogs stained with Sirius red (x40). (F) Representative skeletal muscle (Biceps femoris) sections from early- or advanced-diseased GRMD dogs and age-matched WT stained with sirius red (x40).

Figure S2: NAM supplementation modestly improves skeletal muscle function. (A) Relative tetanic force at 50 Hz in the three treated GRMD dogs before and after one month of NAM treatment (n=3), relative to age-matched WT (n=3) and untreated GRMD dogs (n=8). (B) Half-relaxation time after a tetanic contraction measured during in vivo force measurement test, in the three treated dogs (n=3), before treatment and after one month of NAM treatment, relative to age-matched WT (n=3) and untreated GRMD dogs (n=8). 

Figure S3: Histopathology analysis and serum biomarkers. (A) Percentage of endomysial fibrosis quantified on biceps femoris muscle biopsies from 6 month-old Healthy (n=3), GRMD (n=7) and NAM-treated GRMD (n=3) dogs. ****, P-value<0.00005 (B) Histopathology index quantified on biceps femoris muscle biopsies from 6 month-old Healthy (n=3), GRMD (n=7) and NAM-treated GRMD (n=3) dogs. **, P-value<0.005 (C) Serum creatine kinase (Ck) measured at 6 months of age in Healthy (n=3), GRMD (n=6) and NAM-treated GRMD (n=3) dogs. **, P-value<0.005 (D) Serum myostatin concentration measured at 6 months of age in Healthy (n=3), GRMD (n=5) and NAM-treated GRMD (n=3) dogs. ****, P-value<0.00005 

Figure S4: Uncropped images of membranes stained with Ponceau.
